# Efficacy and safety of intravenous immunoglobulin in systemic sclerosis: a systematic review and meta-analysis

**DOI:** 10.3389/fimmu.2026.1949145

**Published:** 2026-10-05

**Authors:** Jozélio Freire de Carvalho, Roberto Paulo Correia de Araujo, Maria Giovanna Danieli, Yehuda Shoenfeld

**Affiliations:** 1 Institute of Health Sciences, Federal University of Bahia, Salvador, Bahia, Brazil; 2 SOS Immunologia delle Malattie Rare e dei Trapianti, SOD Clinica Medica, Dipartimento di Medicina Interna, Azienda Ospedaliero Universitaria delle Marche, Ancona, Italy; 3 Department of Clinical and Molecular Sciences, Marche Polytechnic University, Ancona, Italy; 4 Reichman University, Herzelia, Israel; 5 Center for Autoimmune Diseases, Sheba Medical Center, Ramat Gan, Israel

**Keywords:** inflammatory myopathy, intravenous immunoglobulin, scleroderma, skin fibrosis, systemic sclerosis

## Abstract

**Background:**

Systemic sclerosis (SSc) is a complex autoimmune disease mainly characterized by progressive fibrosis affecting the skin and internal organs. Although several immunosuppressive and targeted therapies are currently available, many patients continue to experience refractory manifestations. Intravenous immunoglobulin (IVIG) has been increasingly used as an adjunctive treatment in selected cases; however, its efficacy across different disease manifestations remains incompletely defined.

**Objective:**

To systematically evaluate the efficacy and safety of IVIG in patients with systemic sclerosis and to quantitatively synthesize available evidence regarding its effects on skin involvement, gastrointestinal manifestations, and inflammatory myopathy.

**Methods:**

A systematic review and meta-analysis were conducted according to PRISMA 2020 guidelines. MEDLINE/PubMed, Embase, LILACS, and the Cochrane Library were searched from January 1966 to May 2026. Studies evaluating IVIG in adult patients with systemic sclerosis were included. Randomized controlled trials, observational cohorts, pilot studies, and case series were eligible. Quantitative synthesis was performed using random-effects models and standardized mean differences (SMDs) with 95% confidence intervals (CIs).

**Results:**

Eleven studies were included in the systematic review. Six studies provided sufficient quantitative data for the meta-analysis of skin involvement, three for gastrointestinal outcomes, and two for muscle outcomes. The most common indications for IVIG therapy were cutaneous fibrosis, inflammatory myopathy, gastrointestinal involvement, and refractory musculoskeletal manifestations. Meta-analysis demonstrated a significant improvement in skin involvement assessed by the modified Rodnan Skin Score (SMD −0.59, 95% CI −0.95 to −0.22; p = 0.0019; I^2^ = 78.0%). Gastrointestinal manifestations also improved significantly (SMD −0.73, 95% CI −1.16 to −0.29; p = 0.0012; I^2^ = 0%). For inflammatory myopathies, a limited quantitative synthesis of two studies showed a significant reduction in creatinekinase levels (SMD −0.53, 95% CI −0.80 to −0.26; p < 0.001). Across studies, IVIG was well tolerated, and serious adverse events were uncommon.

**Conclusion:**

IVIG was associated with improvements in several clinical outcomes, particularly in observational studies, including skin fibrosis, gastrointestinal manifestations, and inflammatory myopathies. However, the only randomized placebo-controlled trial did not demonstrate significant short-term improvement in skin involvement at its primary endpoint. The available evidence suggests a potential adjunctive role for IVIG in selected patients.

## Introduction

Systemic sclerosis (SSc) is a complex autoimmune connective tissue disease characterized by immune dysregulation, vasculopathy, and progressive fibrosis affecting the skin and multiple internal organs (1). Although considered a rare disease, SSc remains one of the rheumatic disorders associated with the highest morbidity and mortality rates, primarily due to interstitial lung disease, pulmonary arterial hypertension, gastrointestinal involvement, cardiac manifestations, and severe musculoskeletal complications (2). The heterogeneous clinical presentation and unpredictable disease course continue to pose substantial therapeutic challenges despite recent advances in immunomodulatory and antifibrotic therapies (3).

The pathogenesis of SSc involves a complex interplay among innate and adaptive immune activation, endothelial injury, autoantibody production, microvascular dysfunction, and excessive extracellular matrix deposition (4). Activated B lymphocytes, autoreactive T cells, profibrotic cytokines, and pathogenic autoantibodies contribute to persistent inflammation and progressive tissue fibrosis (5). Increasing evidence suggests that immune-mediated mechanisms play a critical role not only during the early inflammatory phase of the disease but also throughout disease progression, providing a rationale for the use of immunomodulatory therapies (6).

Current treatment strategies are largely organ-specific and focus on controlling inflammation, preventing irreversible damage, and improving quality of life (3). Conventional immunosuppressive agents, including mycophenolate mofetil, cyclophosphamide, methotrexate, azathioprine, and rituximab, are commonly employed in selected clinical scenarios (7–10). However, many patients continue to experience progressive skin thickening, refractory musculoskeletal manifestations, severe gastrointestinal dysmotility, inflammatory myopathy, or intolerance to standard therapies. Consequently, alternative therapeutic approaches remain an unmet clinical need in this population.

Intravenous immunoglobulin (IVIG) is a purified preparation of pooled immunoglobulin G obtained from healthy donors and has demonstrated efficacy across a broad spectrum of autoimmune and inflammatory disorders (11). The mechanisms of action of IVIG are multifactorial and include modulation of Fc receptor signaling, neutralization of pathogenic autoantibodies, inhibition of complement activation, regulation of cytokine production, suppression of autoreactive B-cell responses, expansion of regulatory T cells, and attenuation of inflammatory pathways (12, 13). These immunomodulatory properties provide a strong biological rationale for its potential use in systemic sclerosis. Moreover, Rambabu et al. provided new insights into the mechanisms underlying IVIG activity, highlighting its role in modulating metabolic alterations in immune cells (14, 15).

Over the past two decades, IVIG has been increasingly used in patients with refractory SSc, particularly in those with severe cutaneous involvement, inflammatory arthritis, SSc-associated muscle involvement, including reported overlap myositis, gastrointestinal manifestations, and progressive disease despite conventional treatment (11–13). Several observational studies, case series, cohort studies, and a limited number of controlled trials have reported improvements in skin fibrosis, functional outcomes, muscle involvement, and gastrointestinal symptoms, as well as reductions in glucocorticoid exposure in selected patients with associated inflammatory manifestations, particularly myopathy. Nevertheless, the overall body of evidence remains fragmented, and the magnitude of treatment benefit has not been clearly established.

Recent systematic reviews have summarized the available literature regarding IVIG use in SSc. However, these reviews primarily provided qualitative assessments and highlighted the heterogeneity of study designs, treatment regimens, and outcome measures. To date, the quantitative synthesis of available evidence remains limited, and uncertainty persists regarding the efficacy and safety profile of IVIG across different clinical manifestations of systemic sclerosis.

Given the growing interest in IVIG as a potential therapeutic option for refractory SSc and the emergence of additional observational cohorts in recent years, a comprehensive quantitative evaluation of the available evidence is warranted. Therefore, the aim of this systematic review and meta-analysis was to evaluate the efficacy and safety of IVIG therapy in patients with systemic sclerosis, with particular emphasis on cutaneous, musculoskeletal, gastrointestinal, and global clinical outcomes.

## Methods

### Study design and reporting standards

This systematic review and meta-analysis was conducted in accordance with the Preferred Reporting Items for Systematic Reviews and Meta-Analyses (PRISMA 2020) statement (16) and the recommendations of the Cochrane Handbook for Systematic Reviews of Interventions (17). The study protocol was developed *a priori*, including predefined eligibility criteria, outcomes of interest, methods for risk of bias assessment, and statistical analyses. The protocol was not prospectively registered in PROSPERO or another systematic review registry. The review aimed to comprehensively evaluate the efficacy and safety of IVIG therapy in patients with systemic sclerosis across different disease manifestations.

### Literature search strategy

A comprehensive literature search was performed in MEDLINE/PubMed, Embase, LILACS, and the Cochrane Library from January 1966 to May 2026. The search strategy combined controlled vocabulary terms and free-text keywords related to systemic sclerosis and IVIG therapy. Search terms included “systemic sclerosis, “ “scleroderma, “ “diffuse cutaneous systemic sclerosis, “ “limited systemic sclerosis, “ “CREST syndrome, “ “intravenous immunoglobulin, “ “IVIG, “ “immunoglobulin, “ and “gammaglobulin, “ combined using Boolean operators. No restrictions regarding language, publication date, or geographic location were applied. To maximize study identification, the reference lists of all eligible articles, review papers, and relevant guidelines were manually screened for additional studies not retrieved through the electronic search.

### Eligibility criteria

Studies were considered eligible if they included adult patients diagnosed with systemic sclerosis according to recognized classification criteria according to the date on which the studies were conducted or published and evaluated the use of IVIG as a therapeutic intervention. Because the included studies spanned different periods, the specific classification criteria used in each study were recorded when reported, recognizing that historical differences in SSc classification may influence patient selection and contribute to clinical heterogeneity. Both diffuse and limited forms of systemic sclerosis were considered eligible.Randomized controlled trials, non-randomized controlled studies, prospective and retrospective cohort studies, and all original studies regardless of sample size were included. Studies were required to report clinical, functional, laboratory, or safety outcomes associated with IVIG therapy. Publications consisting exclusively of narrative reviews, editorials, expert opinions, letters without original data, conference abstracts lacking sufficient information for extraction, animal studies, and *in vitro* investigations were excluded. Original clinical studies, including case series comprising at least two patients, were eligible for qualitative synthesis when they provided relevant clinical outcome data. Single-patient case reports were excluded because of their very limited generalizability and inability to contribute meaningfully to evidence synthesis. However, only studies with extractable quantitative data were included in meta-analyses.

### Study selection

Two investigators independently screened all titles and abstracts identified through the search strategy. Studies considered potentially relevant underwent full-text evaluation. Eligibility was assessed independently according to the predefined inclusion and exclusion criteria. Any disagreements arising during study selection were resolved through discussion and consensus. When consensus could not be reached, a third reviewer was consulted. The entire selection process was documented using a PRISMA flow diagram (16).

### Data extraction

Data extraction was performed independently by two reviewers using a standardized data collection form. Information obtained from each study included publication characteristics, study design, country of origin, sample size, demographic data, disease subtype, disease duration, SSc classification criteria when reported, indications for IVIG treatment, dosing regimens, duration of therapy, concomitant medications, follow-up duration, efficacy outcomes, and the number and type of adverse events and serious adverse events, when available. Whenever numerical data were reported in graphical format only, values were extracted using validated digital extraction methods. When studies reported medians and interquartile ranges instead of means and standard deviations, established statistical conversion methods were applied whenever appropriate (17).

### Outcomes of interest

The primary outcome of the meta-analysis was the change in skin involvement measured by the modified Rodnan Skin Score (mRSS), which represents the most widely accepted and validated measure of cutaneous fibrosis in systemic sclerosis. When available, mean differences between baseline and post-treatment evaluations were extracted.

Secondary outcomes included changes in gastrointestinal manifestations, muscular involvement, pulmonary function parameters, patient-reported outcomes, changes in glucocorticoid exposure in patients receiving glucocorticoids for associated inflammatory manifestations, global clinical improvement, treatment discontinuation, adverse events, and serious adverse events. Given the multisystemic nature of systemic sclerosis, all clinically relevant manifestations potentially influenced by IVIG therapy were considered during data extraction and synthesis.

### Quality assessment and risk of bias

The methodological quality of included studies was assessed according to study design. Randomized controlled trials were evaluated using the Cochrane Risk of Bias 2 (RoB 2) tool (17). Observational cohort studies were assessed using the Newcastle–Ottawa Scale (NOS), which evaluates participant selection, comparability of study groups, and outcome assessment. Case series were evaluated using the Joanna Briggs Institute Critical Appraisal Checklist for Case Series. Quality assessments were performed independently by two reviewers, with disagreements resolved through consensus. Risk of bias in non-randomized studies was interpreted according to the ROBINS-I framework (18).

### Statistical analysis

Quantitative synthesis was conducted whenever sufficient homogeneous data were available. Considering the anticipated clinical and methodological heterogeneity among studies, random-effects models were selected as the primary analytical approach (17). Continuous outcomes were pooled using mean differences or standardized mean differences with corresponding 95% confidence intervals. Dichotomous outcomes were summarized using pooled proportions, odds ratios, or risk ratios, depending on the nature of the reported data.

Statistical heterogeneity was evaluated using Cochran’s Q test and quantified with the I^2^ statistic. Values of approximately 25%, 50%, and 75% were interpreted as low, moderate, and high heterogeneity, respectively (17). When substantial heterogeneity was identified, potential sources were explored through subgroup and sensitivity analyses.

Prespecified subgroup analyses included comparisons according to disease subtype, presence of overlap myositis, gastrointestinal involvement, IVIG treatment indication, study design, and duration of follow-up, when sufficient study-level data were available to permit meaningful stratification. Sensitivity analyses were performed through sequential exclusion of individual studies and exclusion of studies considered at high risk of bias.

Because fewer than ten studies contributed to each individual pooled outcome, formal assessment of small-study effects and publication bias using funnel plots or statistical tests for funnel-plot asymmetry was not performed because such analyses would have limited power and potentially unreliable interpretation. Statistical analyses were performed using Review Manager (RevMan) version 5.4 and R statistical software. A two-sided p value below 0.05 was considered statistically significant for all analyses. The certainty of evidence was interpreted according to the GRADE framework (19).

## Results

### Study selection

The literature search identified studies evaluating the efficacy and safety of IVIG in patients with systemic sclerosis. After removal of duplicates and screening of titles and abstracts, potentially relevant articles underwent full-text assessment. A total of 11 primary studies fulfilled the eligibility criteria for the systematic review, comprising one randomized controlled trial, several retrospective and prospective observational cohorts, pilot studies, and case series (20–30). Three systematic reviews were identified and used for bibliographic screening and contextual discussion but were not included in the quantitative synthesis (31–34). The study selection process is summarized in **Figure 1**.

**Figure 1:**
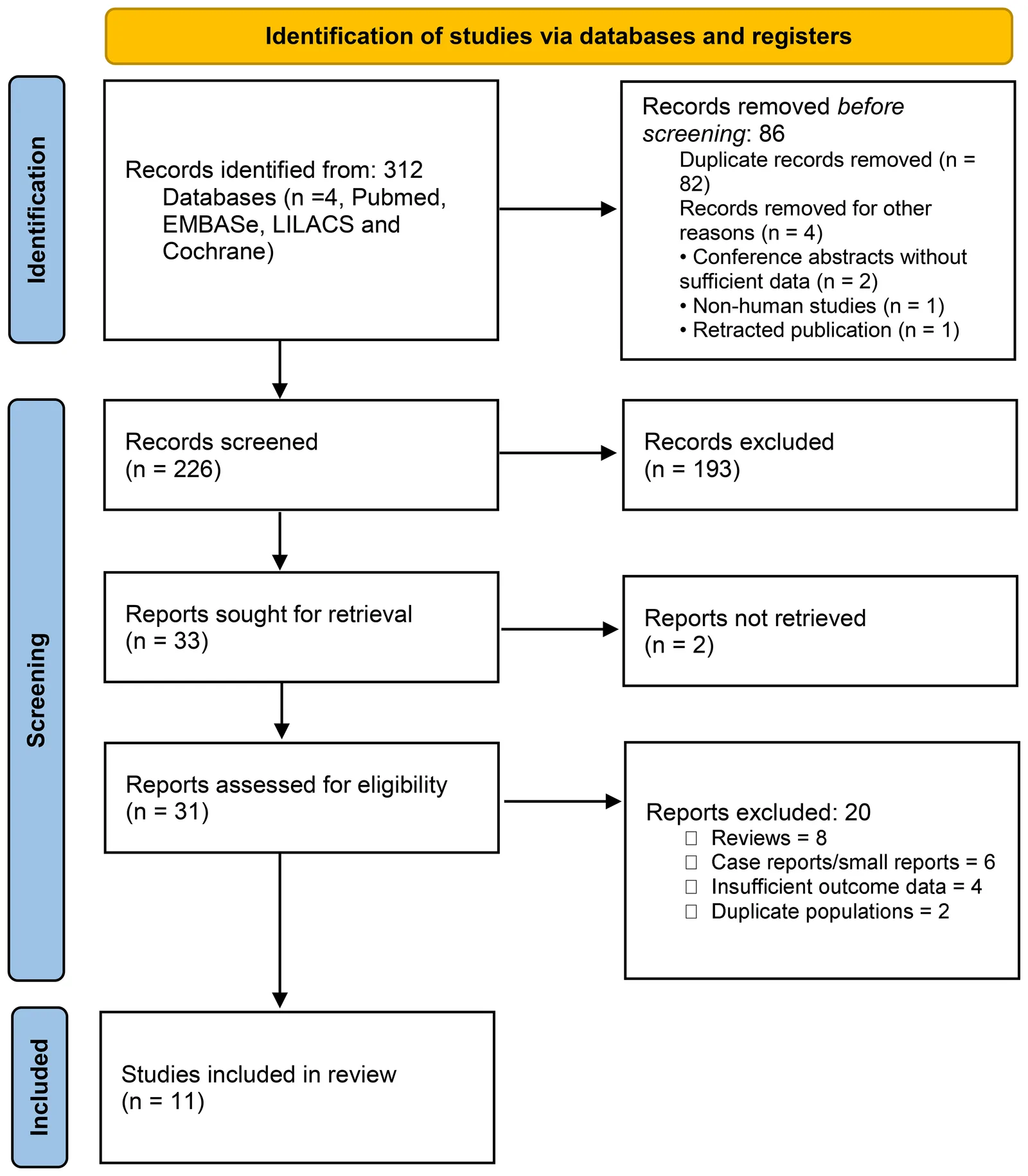
PRISMA 2020 flow diagram of study selection process.

### Characteristics of included studies

The included studies were published between 2007 and 2023 and originated from multiple countries, including Japan, France, Italy, Spain, the United Kingdom, and the United States (20–30). Although the literature search covered the period from January 1966 onward, the earliest primary clinical study meeting the eligibility criteria was published in 2007. Most investigations were observational in nature, reflecting the rarity of systemic sclerosis and the off-label use of IVIG in refractory disease manifestations. The main characteristics of the included studies are summarized in **Table 1**.

**Table 1:** Characteristics of primary studies included in the systematic review of intravenous immunoglobulin (IVIG) in systemic sclerosis.

Study [author, year (ref.)]	Country	Study design	N (SSc)	Main SSc phenotype	SSc classification/diagnostic criteria	Main indication for IVIG	IVIG regimen	Follow-up	Main outcomes evaluated
Nacci et al., 2007 (20)	Italy	Pilot study	7	Limited and diffuse SSc	NR	Refractory arthritis	2 g/kg/month × 6 cycles	6 months	HAQ, VAS, mRSS, hand function
Takehara et al., 2013 (21)	Japan	Randomized double-blind placebo-controlled trial	63	Diffuse cutaneous SSc	NR; study eligibility required dcSSc with mRSS ≥20	Skin sclerosis	400 mg/kg/day × 5 days	60 weeks	mRSS
Poelman et al., 2015 (22)	USA	Retrospective cohort	30	Diffuse cutaneous SSc	LeRoy et al. classification for the diffuse cutaneous subset	Refractory skin disease	2 g/kg/month	24 months	mRSS
Clark et al., 2015 (23)	UK	Case series	2	SSc-myositis overlap	NR	Gastrointestinal involvement in SSc-myositis overlap	2 g/kg over 5 days	5 and 11.5 months	UCLA SCTC GIT 2.0, gastrointestinal symptoms, muscle involvement
Raja et al., 2016 (24)	United Kingdom	Observational cohort	15	SSc-overlap myositis	2013 ACR/EULAR criteria	Gastrointestinal involvement	IVIG	Mean 2.3 years	UCLA SCTC GIT 2.0, mRSS, muscle strength
Sanges et al., 2017 (25)	France	Nationwide multicenter cohort	46	Mixed	2013 ACR/EULAR criteria; cutaneous subsets classified according to LeRoy et al.	Multiple refractory manifestations	IVIG	Variable	Skin, muscle, GI, safety
Chaigne et al., 2020 (26)	France	Comparative retrospective study	52	Limited and diffuse SSc	2013 ACR/EULAR criteria	SSc-associated myopathy	IVIG adjunctive therapy	Variable	Corticosteroid-sparing effect, muscle outcomes
Mendoza et al., 2022 (27)	USA	Case series	2	SSc with severe dysphagia	2013 ACR/EULAR criteria	Swallowing dysfunction	2 g/kg/month for 6 months	6 months	UCLA SCTC GIT 2.0, esophageal motility
Matsuda et al., 2023 (28)	Japan	Case series	3	SSc-myositis overlap	NR	Intestinal pseudo-obstruction	IVIG	Short-term	Gastrointestinal improvement
Tandaipan et al., 2023 (29)	Spain	Multicenter retrospective cohort	78	59% diffuse SSc	2013 ACR/EULAR criteria	Myositis, GI and skin involvement	IVIG	Variable	mRSS, MRC, CK, UCLA SCTC GIT 2.0
Bonomi et al., 2023 (30)	Italy	Retrospective cohort (ACR abstract)	65	Mixed	2013 ACR/EULAR criteria	Gastrointestinal, cutaneous and vascular involvement	2 g/kg/month	12 months	mRSS, digital ulcers, edema, GI manifestations

ACR, American College of Rheumatology; CK, creatine kinase; EULAR, European Alliance of Associations for Rheumatology; GI, gastrointestinal; HAQ, Health Assessment Questionnaire; IVIG, intravenous immunoglobulin; MRC, Medical Research Council muscle strength scale; mRSS, modified Rodnan Skin Score; NR, not reported; SSc, systemic sclerosis; UCLA SCTC GIT 2.0, University of California Los Angeles Scleroderma Clinical Trials Consortium Gastrointestinal Tract 2.0 questionnaire; VAS, visual analogue scale.

Systematic reviews identified during the search process (Garrote-Corral et al. (32), Koczanowski et al. (33), and Neto et al. (34)) were used for bibliographic screening and contextual discussion but were not considered primary studies and were therefore not included in **Table 1**.

Overall, more than 350 patients with systemic sclerosis received IVIG across the included primary studies. Sample sizes ranged from 2 to 78 patients, with women accounting for the majority of participants in all cohorts (20–30). The mean age of participants ranged from approximately 52 to 59 years, and both diffuse cutaneous systemic sclerosis (dcSSc) and limited cutaneous systemic sclerosis (lcSSc) were represented. However, diffuse cutaneous disease predominated in most studies.

IVIG was generally administered at a dose of 2 g/kg per cycle, divided over 2 to 5 consecutive days and repeated monthly (20–30). Treatment duration varied substantially among studies, ranging from a single treatment cycle to more than 24 months of follow-up. Most patients received concomitant immunosuppressive therapies, including glucocorticoids, mycophenolate mofetil, methotrexate, azathioprine, cyclophosphamide, or biologic agents. The number of IVIG courses (29) varied substantially across studies and no single median number of courses associated with clinical response could be established. For example, the median number of courses was 6 (2–16) in Chaigne et al. (26) and 5 (range 1–60) in Tandaipan et al. (29), whereas Nacci et al. (20) administered six courses and Poelman et al. (22) reported 3–24 monthly cycles.

The indications for IVIG administration were heterogeneous. The most frequent indications included cutaneous fibrosis, inflammatory myopathy associated with systemic sclerosis, gastrointestinal involvement, refractory musculoskeletal manifestations, and overlap syndromes, predominantly overlap with idiopathic inflammatory myopathy (20–30). In the largest multicenter cohort, myositis represented the most common indication for treatment, followed by gastrointestinal and cutaneous involvement (35). The quantitative data extracted for the meta-analysis, including sample size, treatment regimen, follow-up duration, and outcome measures, are presented in **Table 2**.

**Table 2:** Quantitative studies included in the meta-analyses.

Outcome	Study [author, year (ref.)]	N	Assessment tool	Baseline value	Post-IVIG value	Follow-up	Included in meta-analysis
Skin involvement	Nacci et al., 2007 (20)	7	mRSS	29.2 ± 8.3	21.1 ± 4.6	6 months	Yes
Skin involvement	Takehara et al., 2013 (21)	63	mRSS	Reported in study	Reported in study	60 weeks	Yes
Skin involvement	Poelman et al., 2015 (22)	30	mRSS	29.6 ± 7.2	22.5 ± 10.0	12 months	Yes
Skin involvement	Raja et al., 2016 (24)	15	mRSS	21.5 ± 13.8	10.0 ± 10.6	Mean 2.3 years	Yes
Skin involvement	Sanges et al., 2017 (25)	46	mRSS	No significant change	No significant change	Variable	Yes
Skin involvement	Tandaipan et al., 2023 (29)	78	mRSS	15.0 ± 12.4	13.0 ± 12.5	Variable	Yes
Gastrointestinal involvement	Raja et al., 2016 (24)	15	UCLA SCTC GIT 2.0	Reported in study	Improved after IVIG	Mean 2.3 years	Yes
Gastrointestinal involvement	Mendoza et al., 2022 (27)	2	UCLA SCTC GIT 2.0/Esophageal assessment	Severe baseline dysfunction	Marked improvement	6 months	Yes
Gastrointestinal involvement	Tandaipan et al., 2023 (29)	78	UCLA SCTC GIT 2.0	1.06 ± 0.61	0.78 ± 0.40	Variable	Yes
Inflammatory myopathy	Sanges et al., 2017 (25)	46	Serum CK	Reported in study	Improved after IVIG	Variable	Yes
Inflammatory myopathy	Tandaipan et al., 2023 (29)	78	Serum CK	1149 ± 2026 U/L	217 ± 224 U/L	Variable	Yes

CK, creatine kinase; IVIG, intravenous immunoglobulin; mRSS, modified Rodnan Skin Score; N, number of patients; SCTC, Scleroderma Clinical Trials Consortium; UCLA, University of California Los Angeles.

### Cutaneous outcomes

Skin involvement, assessed primarily using the modified Rodnan Skin Score (mRSS), was the most frequently evaluated outcome. Several observational studies reported significant reductions in mRSS following IVIG therapy (20, 22, 24, 29).

In the cohort reported by Poelman et al. (22), mean mRSS decreased from 29.6 ± 7.2 at baseline to 24.1 ± 9.6 and 22.5 ± 10.0 at 12 months. Similar improvements were reported by Raja et al. (24), who observed a reduction from 21.5 ± 13.8 before treatment to 10.0 ± 10.6 after treatment.

Nacci et al. (20) also demonstrated significant improvement in skin scores after six months of therapy, with mRSS decreasing from 29.2 ± 8.3 to 21.1 ± 4.6. Likewise, Tandaipan et al. (29) reported a significant reduction in mRSS from 15.0 ± 12.4 to 13.0 ± 12.5 following treatment.

The only randomized double-blind placebo-controlled trial, conducted by Takehara et al. (21), included 63 patients with diffuse cutaneous systemic sclerosis. The primary endpoint was not met: at 12 weeks, the mean change in mRSS was −3.3 ± 4.2 in the IVIG group and −4.2 ± 4.6 in the placebo group, with no significant between-group difference. In the subsequent non-blinded readministration phase, patients receiving two IVIG courses showed a greater reduction in mRSS at week 60 than those receiving placebo followed by one IVIG course (−8.3 ± 1.0 vs −4.1 ± 1.1; p=0.004).

Not all studies demonstrated positive results. In the French nationwide cohort reported by Sanges et al. (25), no statistically significant change in mRSS was observed after treatment. When analyzed according to cutaneous subset, no significant change was observed in either limited cutaneous SSc (8.6 ± 5.1 to 5.9 ± 4.6; p=0.13) or diffuse cutaneous SSc (22.0 ± 9.8 to 22.3 ± 11.5; p=0.79). Nevertheless, the study suggests that IVIg is a safe therapeutic option that may improve musculoskeletal involvement, systemic inflammation, and digestive tract symptoms while also exerting corticosteroid-sparing effect (25).

### Musculoskeletal and myopathy outcomes

Musculoskeletal manifestations represented another important indication for IVIG therapy. Several studies reported improvements in inflammatory arthritis, tendon friction rubs, muscle strength, and biochemical markers of muscle inflammation (20, 24–26, 29).

In the multicenter study by Tandaipan et al. (29), 92% of patients with inflammatory myopathy demonstrated improvement in muscle strength, accompanied by a significant reduction in creatine kinase levels from 1149 ± 2026 U/L to 217 ± 224 U/L. Importantly, clinical muscle function also improved, with the number of patients with Medical Research Council (MRC) muscle strength <3/5 decreasing from 26 to 2 after treatment. Similarly, Raja et al. (24) reported improvements in muscle manifestations, including muscle strength assessed using the Medical Research Council (MRC) Sum Score, in addition to changes in CK levels.

Sanges et al. (25) similarly demonstrated improvement in clinical muscle involvement, with muscle weakness decreasing from 45% to 21% (p=0.01), alongside reductions in muscle pain and CK levels.

Chaigne et al. (26) evaluated 52 patients with systemic sclerosis-associated myopathy, including 18 treated with IVIG, and demonstrated greater glucocorticoid dose reduction among IVIG-treated patients. The median glucocorticoid dose decreased by 5 [0–12.5] mg/day at month 3 after IVIG initiation compared with 0 [0–2.5] mg/day in patients not receiving IVIG (p<0.05), and the median dose at one year was 5 [5–5.75] versus 7.5 (5–10) mg/day, respectively (p<0.05). Although skin outcomes were not significantly different between treatment groups, improvements in muscle disease control allowed meaningful reductions in glucocorticoid exposure.

### Gastrointestinal outcomes

Gastrointestinal involvement was assessed in several studies using symptom-based evaluations and the UCLA SCTC GIT 2.0 questionnaire (24, 27–29). Improvements in gastrointestinal manifestations were consistently reported across studies.

Tandaipan et al. (29) demonstrated a reduction in total UCLA GIT 2.0 scores from 1.06 ± 0.61 before treatment to 0.78 ± 0.40 after treatment. Raja et al. (24) similarly reported significant improvements in gastrointestinal symptom burden. Clark et al. reported two patients with SSc-myositis overlap syndrome and severe gastrointestinal involvement; UCLA SCTC GIT 2.0 scores decreased from 2.75 to 2.05 after 11.5 months in one patient and from 1.69 to 1.25 within 5 months in the other (23). Additional case series involving severe dysphagia and intestinal pseudo-obstruction described clinically meaningful improvements following IVIG administration, suggesting a potential role for immunomodulation in selected gastrointestinal manifestations of systemic sclerosis (27, 28).

### Pulmonary outcomes

Pulmonary outcomes were evaluated less consistently. Across studies reporting pulmonary function tests, forced vital capacity (FVC) and diffusing capacity for carbon monoxide (DLCO) generally remained stable throughout follow-up (22, 25, 26, 29). Although significant improvements were not consistently observed, stabilization of pulmonary disease was frequently reported.

### Safety

IVIG therapy was generally well tolerated. In the French nationwide cohort of 46 patients, two severe adverse events were reported (one deep-vein thrombosis and one diffuse edematous syndrome), both leading to IVIG discontinuation; minor events included three cases each of fever/chills, skin rash, and headache. No acute renal failure occurred during treatment (25).

In Chaigne et al. (26), 8/18 IVIG-treated patients (44%) experienced at least one adverse event, including three patients with serious adverse events: two cases of acute kidney injury and one case of aseptic meningitis. The two patients with acute kidney injury discontinued IVIG, and no IVIG-related deaths were observed.

In Poelman et al. (22), the most frequent adverse events were headache (40%), fatigue (23.3%), nausea with or without vomiting (13.3%), fever (10%), arthralgia/myalgia (10%), rash/pruritus (10%), and generalized weakness (10%). One patient discontinued treatment because of aseptic meningitis and another because of a transient ischemic attack.

Overall, adverse-event reporting was heterogeneous across studies, precluding a reliable pooled incidence estimate. **Table 3** summarizes study-level safety data, including denominators and reported counts of any adverse events, serious adverse events, and adverse event–related treatment discontinuations; heterogeneity in adverse-event definitions and reporting precluded calculation of pooled incidence estimates.

**Table 3:** Safety and adverse events reported with IVIG in systemic sclerosis.

Study	IVIG-treated patients, n	Any adverse events	Serious adverse events	Treatment discontinuation due to adverse events	Specific adverse events reported
Nacci et al. (20)	7	0/7	0 reported	0	No adverse reactions were observed during or after IVIG treatment.
Takehara et al. (21)	31 in the randomized IVIG arm	10/31 (32.3%) adverse drug reactions during the double-blind phase	NR	NR	Main reported reactions were fever and elevations in CRP and ALT. Laboratory abnormalities occurred in 8/31 (25.8%). In the readministration phase, adverse drug reactions occurred in 7/18 (38.9%) in the IVIG→IVIG group and 6/19 (31.6%) in the placebo→IVIG group.
Poelman et al. (22)	30	Reported by event: headache 12/30 (40%); fatigue 7/30 (23.3%); nausea/emesis 4/30 (13.3%); fever 3/30 (10%); arthralgia/myalgia 3/30 (10%); rash/pruritus 3/30 (10%); generalized weakness 3/30 (10%); hypertension 2/30 (6.7%)	1 aseptic meningitis; 1 transient ischemic attack led to discontinuation. An acute kidney injury occurred in one patient 15 months after IVIG had already been discontinued and should not be counted as an on-treatment IVIG SAE.	2/30 specifically reported as stopping because of aseptic meningitis or TIA	See event counts at left. One death from sudden cardiac arrest occurred in a patient with pre-existing cardiomyopathy and was not described as IVIG-related.
Raja et al. (24)	15	No significant side effects reported	0 significant SAEs reported	0 reported	Authors state that IVIG was well tolerated in all 15 patients without significant side effects.
Sanges et al. (25)	46	Minor events: fever/chills 3 cases; skin rash 3 cases; headache 3 cases	2/46 severe events: 1 deep-vein thrombosis and 1 diffuse edematous syndrome	3/46 (7%) discontinued because of adverse events; both severe events led to discontinuation	No acute renal failure was reported during treatment. The article reports 3 discontinuations due to adverse events overall, although only 2 events were classified as severe.
Chaigne et al. (26)	18	8/18 (44%) had ≥1 AE	3/18 (16.7%): 2 acute kidney injuries and 1 aseptic meningitis	2/18 (11.1%), both because of acute kidney injury	Mild/moderate events occurred in 5 patients during the first infusion: fever n=3, hypotension n=1, abdominal pain n=1, urticarial rash n=1, headache n=1. No IVIG-related deaths.
Mendoza et al. (27)	2	1/2 had low-grade fever during infusion	0/2 severe adverse events attributable to IVIG	0	Patient 1 had no adverse events; patient 2 had low-grade fever after administration.
Matsuda et al. (28)	3	1 event: frequent defecation (n=1)	None reported in the three-case series	NR	In the authors’ own three cases, the table reports no severe adverse events and frequent defecation in one patient.
Tandaipan et al. (29)	78	12 adverse events in 10/78 patients	1 serious adverse effect leading to withdrawal: severe hemolysis	4/78 withdrew after adverse events; only 1 withdrawal was attributed to a serious AE	Events: systemic hypertension n=3; headache n=3; oral candidiasis n=2; hemolytic anemia n=1; thrombocytopenia n=1; reflex sympathetic dystrophy syndrome n=1; traumatic hemorrhagic shock n=1. No AE-related deaths.
Bonomi et al. (30)	55 IV, within a cohort of 65 receiving IV or SC Ig	NR	NR	NR	The conference abstract reports efficacy outcomes but does not provide safety/adverse-event data. Fifty-five of 65 patients received immunoglobulin intravenously.

AE, adverse event; ALT, alanine aminotransferase; CRP, C-reactive protein; IVIG, intravenous immunoglobulin; NR, not reported; SAE, serious adverse event; TIA, transient ischemic attack.

### Risk of bias

The overall quality of evidence was limited by the predominance of observational studies, small sample sizes, lack of control groups, and heterogeneity in treatment regimens and outcome assessment (20–30). Only one randomized controlled trial was identified (21). Consequently, the certainty of evidence was considered low to moderate for most outcomes, supporting the need for quantitative synthesis and cautious interpretation of pooled estimates. The detailed risk-of-bias assessment is presented in **Table 4**.

**Table 4:** Risk of bias assessment of included studies.

Study	Design	Tool	Main concerns	Overall risk of bias
Takehara 2013 (21)	Randomized double-blind placebo-controlled trial	RoB 2	Small sample, short double-blind phase, complex extension/readministration design	High
Nacci 2007 (20)	Pilot before-after study	ROBINS-I/JBI	No control group, small sample, confounding by concomitant therapies	Serious
Poelman 2015 (22)	Retrospective cohort	ROBINS-I	No randomized comparator, historical controls, concomitant immunosuppression	Serious
Raja 2016 (24)	Observational cohort	ROBINS-I	No control group, overlap myositis population, concomitant therapies	Serious
Sanges 2017 (25)	Multicenter retrospective cohort	ROBINS-I	Heterogeneous indications, no control group, concomitant immunosuppression	Serious
Chaigne 2020 (26)	Comparative retrospective cohort	ROBINS-I	Non-randomized treatment allocation, confounding by disease severity and corticosteroid exposure	Serious
Tandaipan 2023 (29)	Multicenter retrospective cohort	ROBINS-I	Heterogeneous indications, no control group, concomitant therapies	Serious
Bonomi 2023 (30)	Retrospective cohort/meeting abstract	ROBINS-I	Abstract-only data, incomplete reporting, no control group	Critical
Mendoza 2022 (27)	Case series	JBI Case Series	Two patients only, no comparator, high selection bias	Critical
Matsuda 2023 (28)	Case series + review	JBI Case Series	Very small sample, short-term outcomes, no comparator	Critical

DB, double-blind, JBI, Joanna Briggs Institute, ROBINS-I, Risk Of Bias In Non-randomized Studies of Interventions, RoB 2, Risk of Bias 2 tool, RCT, randomized controlled trial, SSc, systemic sclerosis.

### Meta-analysis of skin involvement

Six studies provided sufficient quantitative data on skin involvement assessed by the modified Rodnan Skin Score (mRSS) and were included in the primary meta-analysis (20–22, 24, 25, 29). Given the variability in outcome reporting and follow-up duration across studies, pooled analyses were performed using standardized mean differences (SMDs) and a random-effects model.

IVIG therapy was associated with a significant improvement in skin fibrosis, with a pooled SMD of −0.59 (95% CI −0.95 to −0.22; p = 0.0019), corresponding to a moderate treatment effect favoring IVIG (**Figure 2**). Substantial heterogeneity was observed among studies (I^2^ = 78.0%), suggesting important clinical and methodological variability.

**Figure 2:**
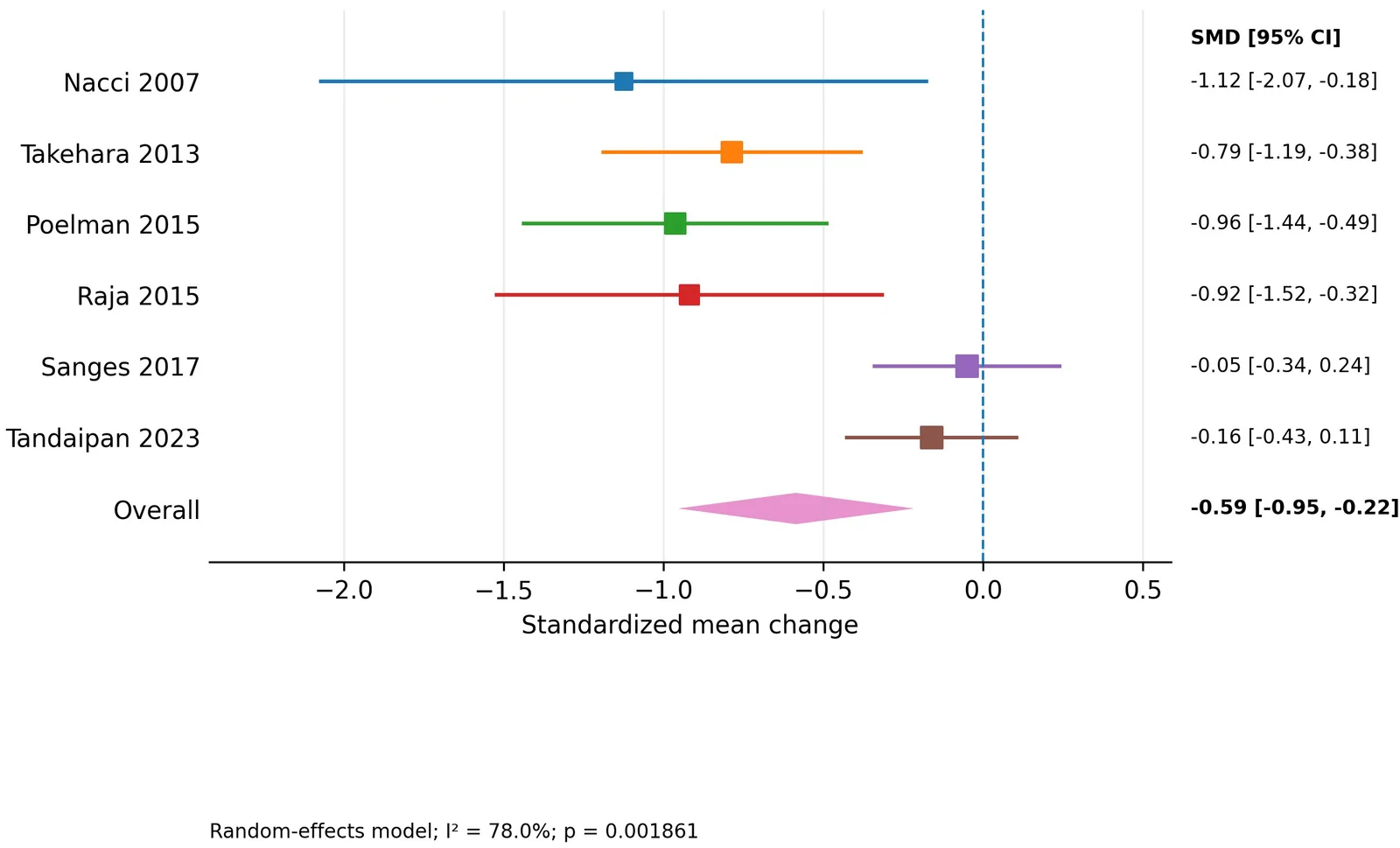
Forest plot of the effect of intravenous immunoglobulin on skin involvement assessed by modified Rodnan Skin Score (mRSS).

Potential sources of heterogeneity included differences in disease duration, baseline skin severity, proportions of diffuse cutaneous systemic sclerosis, concomitant immunosuppressive therapies, IVIG regimens, and follow-up duration. Despite this variability, sensitivity analyses demonstrated a consistent direction of effect favoring IVIG across studies. Available study-level data did not permit a robust pooled subgroup comparison between limited and diffuse cutaneous SSc. A formal subgroup meta-analysis according to study design was also not considered statistically informative because only one randomized placebo-controlled trial contributed to the cutaneous analysis, whereas the remaining studies were observational.

In the Sanges cohort, the only study providing directly stratified mRSS outcomes, neither lcSSc nor dcSSc showed a statistically significant change in mRSS (25).

The randomized placebo-controlled trial by Takehara et al. (21) did not demonstrate a significant between-group difference in mRSS during the 12-week double-blind phase. A greater reduction in mRSS was subsequently observed among patients receiving two IVIG courses compared with those receiving placebo followed by a single IVIG course during the non-blinded readministration phase; this latter finding should therefore be interpreted separately from the randomized primary endpoint.

### Meta-analysis of gastrointestinal outcomes

Three studies reported sufficient quantitative data regarding gastrointestinal involvement and were included in the pooled analysis (24, 27, 29). Gastrointestinal manifestations were primarily evaluated using the UCLA SCTC GIT 2.0 instrument.

IVIG treatment was associated with significant improvement in gastrointestinal symptoms, with a pooled SMD of −0.73 (95% CI −1.16 to −0.29; p = 0.0012), representing a moderate-to-large treatment effect (**Figure 3**). No significant statistical heterogeneity was detected among studies (I^2^ = 0%).

**Figure 3:**
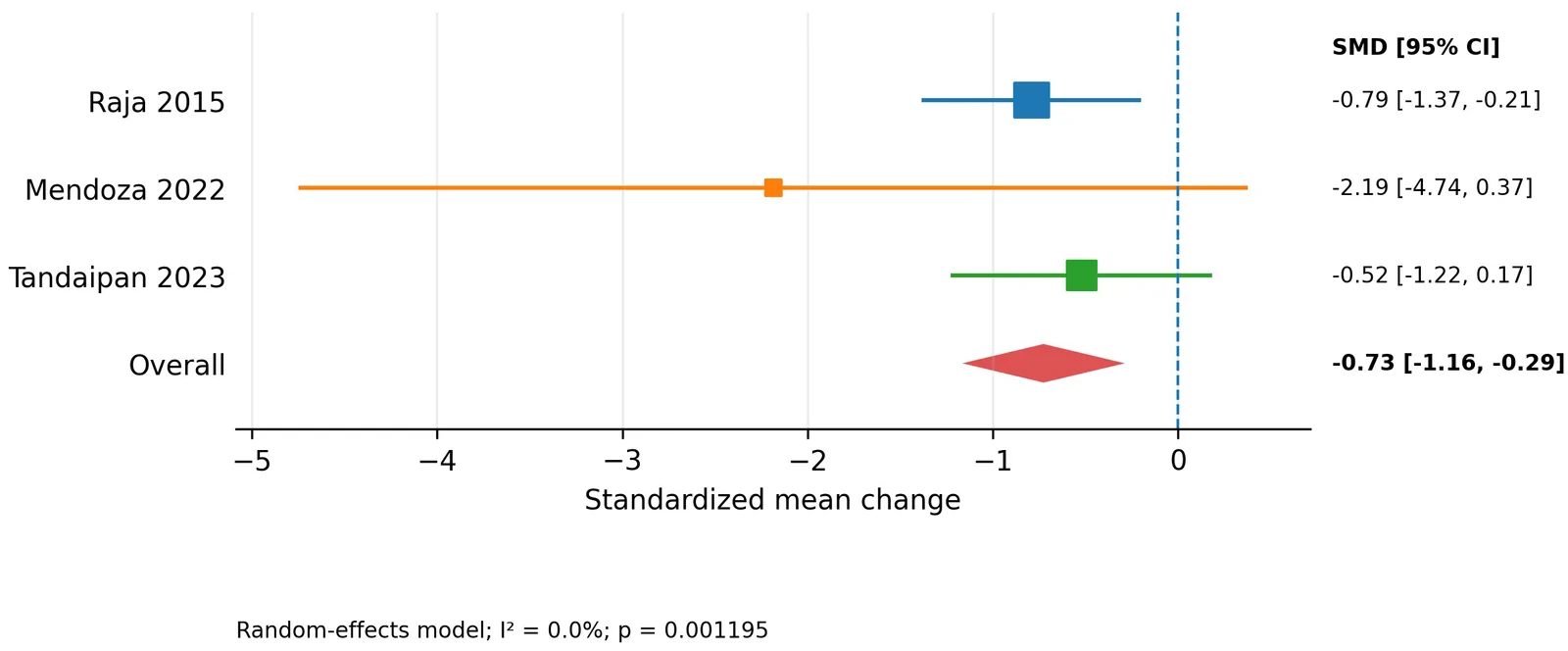
Forest plot of the effect of intravenous immunoglobulin on gastrointestinal involvement assessed by UCLA SCTC GIT 2.0.

Clinical improvements were most evident among patients presenting with severe gastrointestinal dysmotility, dysphagia, intestinal pseudo-obstruction, and upper gastrointestinal manifestations. The consistency of the findings across studies supports a potential role for IVIG in selected gastrointestinal complications of systemic sclerosis.

### Meta-analysis of muscle outcomes

Two studies evaluating systemic sclerosis-associated inflammatory myopathy provided sufficient quantitative data for pooled analysis using serum creatine kinase (CK) levels as an objective marker of muscle inflammation (25, 29).

IVIG therapy was associated with significant improvement in muscle involvement, with a pooled SMD of −0.53 (95% CI −0.80 to −0.26; p < 0.001), representing a moderate treatment effect (**Figure 4**). No statistical heterogeneity was detected (I^2^ = 0%); however, because only two studies contributed to this analysis, the estimate of between-study heterogeneity is imprecise and should not be interpreted as evidence of true homogeneity.

**Figure 4:**
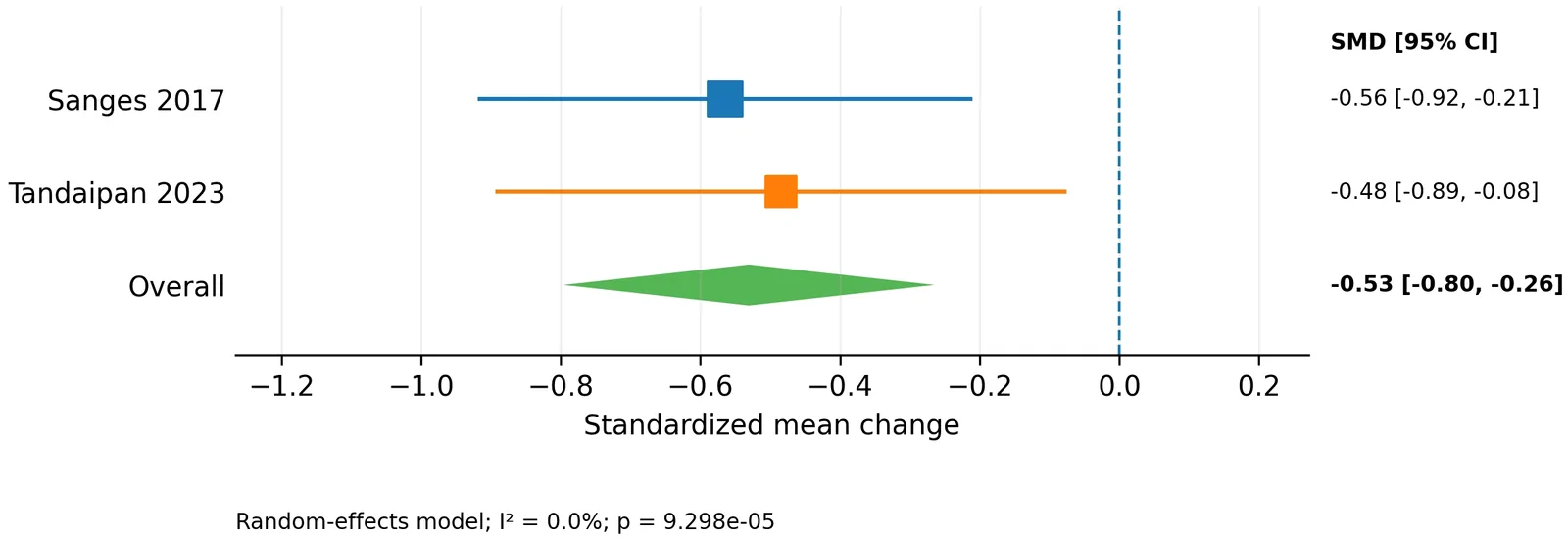
Forest plot of the effect of intravenous immunoglobulin on muscle involvement assessed by serum creatine kinase (CK) levels.

Furthermore, the pooled analysis was based on CK levels, a biochemical marker of muscle injury, and should not be interpreted as equivalent to improvement in muscle function. Nevertheless, clinical improvement in muscle strength was independently reported in observational cohorts using MRC-based assessments, including Tandaipan et al. (29) and Sanges et al. (25).

These findings were supported by the clinical observations reported in the included cohorts, which consistently demonstrated improvements in muscle strength, reductions in CK concentrations, and meaningful corticosteroid-sparing effects (24, 26, 29).

### Overall interpretation of quantitative findings

Across the pooled analyses, statistically significant associations were observed for skin, gastrointestinal, and muscle outcomes; however, interpretation is limited by the predominance of observational evidence, substantial heterogeneity in the cutaneous analysis, and the small number of studies contributing to gastrointestinal and muscle outcomes. These findings suggest potential clinical signals for IVIG in selected SSc manifestations but should not be interpreted as establishing efficacy.

Although the certainty of evidence remains limited by the predominance of observational studies and the relatively small sample sizes, the observed findings, particularly for gastrointestinal and muscle outcomes, support further investigation of IVIG in selected SSc manifestations rather than its use as an established therapeutic option. The forest plots illustrating the pooled effect estimates for skin, gastrointestinal, and muscle outcomes are presented in **Figures 2**–**4**, respectively.

## Discussion

In this systematic review and meta-analysis, IVIG was associated with significant improvements in the three major domains evaluated in systemic sclerosis (SSc), namely skin fibrosis, gastrointestinal manifestations, and inflammatory myopathy. To our knowledge, this study represents one of the first quantitative syntheses specifically evaluating the efficacy of IVIG across multiple clinical manifestations of SSc. The pooled analyses suggested moderate treatment effects across these domains; however, the certainty of these findings differed substantially according to study design and outcome, with most evidence derived from observational studies. These findings support a possible adjunctive role for IVIG in selected patients with refractory disease (22, 24–26, 29).

Skin involvement was the most frequently evaluated outcome, but the evidence regarding cutaneous efficacy requires cautious interpretation. Cutaneous fibrosis remains one of the hallmarks of systemic sclerosis and is closely associated with disease burden, functional impairment, and long-term prognosis (1, 2). The pooled analysis demonstrated a significant reduction in mRSS following IVIG treatment, corresponding to a moderate effect size. However, substantial heterogeneity was observed (I^2^=78%), and most contributing studies were uncontrolled observational cohorts. Importantly, the only randomized double-blind placebo-controlled trial did not demonstrate a significant difference between IVIG and placebo at the prespecified 12-week primary endpoint. Although the direction of effect favored IVIG across several observational studies (20–24, 29), the pooled result should therefore not be interpreted as establishing short-term cutaneous efficacy. This finding is clinically relevant because mRSS remains the most widely accepted surrogate marker of disease activity and skin fibrosis in systemic sclerosis (1, 3). Moreover, improvements in skin scores have frequently been associated with better physical function, quality of life, and long-term outcomes (22, 24, 29).

The mechanisms through which IVIG may influence skin fibrosis remain incompletely understood but are biologically plausible. Experimental and translational studies have demonstrated that IVIG can modulate both innate and adaptive immune responses, reduce autoantibody-mediated tissue injury, inhibit complement activation, and regulate cytokine networks involved in fibrosis (11–13). Previous studies have suggested that IVIG may interfere with transforming growth factor-beta (TGF-β) signaling, fibroblast activation, and extracellular matrix deposition, pathways that play central roles in systemic sclerosis pathogenesis (4–6). These immunomodulatory effects provide biological plausibility for the clinical observations reported in several studies; however, a causal relationship between repeated IVIG cycles and progressive cutaneous improvement cannot be established from the available uncontrolled data (21, 22, 24).

Beyond its effects on fibrosis, IVIG may exert broader immunological actions that are particularly relevant to systemic sclerosis. Increasing evidence indicates that B-cell hyperactivity, abnormal T-cell responses, and dysregulated innate immune pathways contribute substantially to disease pathogenesis (5, 11–13). IVIG has been shown to modulate Fc receptor signaling, neutralize pathogenic autoantibodies, inhibit complement activation, and promote the expansion of regulatory T cells (11–13, 36, 37). These mechanisms may be especially important in patients presenting with overlap syndromes, inflammatory myopathy, or active immune-mediated manifestations. In the present review, the term “inflammatory manifestations” refers descriptively to clinical settings such as SSc-associated myopathy, inflammatory musculoskeletal involvement, and overlap with idiopathic inflammatory myopathy, rather than to a formally validated SSc inflammatory phenotype. The observation that clinically apparent benefits were reported particularly in these settings is consistent with, but does not prove, a predominantly immunomodulatory mechanism of IVIG (36–40).

Another noteworthy aspect is the potential influence of IVIG on vascular dysfunction, a central feature of systemic sclerosis (1, 2, 4). Endothelial injury represents one of the earliest pathogenic events in the disease and contributes to Raynaud phenomenon, digital ulcers, pulmonary vascular disease, and progressive tissue ischemia (2, 4). Although the studies included in this review did not consistently evaluate vascular outcomes, several investigators reported improvements in peripheral vascular symptoms, digital ulcer healing, and inflammatory edema following IVIG treatment (25, 29, 30). These observations raise the possibility that IVIG may indirectly improve vascular homeostasis through modulation of endothelial inflammation and autoantibody-mediated vascular injury (36, 38). Future studies should specifically investigate vascular endpoints to clarify this potential therapeutic effect.

Another important finding of the present study was the significant improvement in gastrointestinal manifestations. Gastrointestinal involvement affects the majority of patients with systemic sclerosis and represents one of the leading causes of morbidity (1, 2). Dysphagia, gastroesophageal reflux, gastroparesis, intestinal dysmotility, bacterial overgrowth, and pseudo-obstruction substantially impair quality of life and often remain refractory to conventional treatment. The pooled analysis demonstrated a moderate-to-large beneficial effect of IVIG on gastrointestinal outcomes, with remarkably consistent findings across studies (24, 27–29, 34). Although the exact mechanism remains uncertain, these observations support the hypothesis that immune-mediated neuronal and smooth muscle dysfunction may contribute to gastrointestinal manifestations in at least a subset of patients (27, 28, 34). Consequently, immunomodulatory interventions such as IVIG may provide benefit beyond symptomatic management alone (27, 28, 34).

The findings regarding inflammatory myopathy are also noteworthy. Muscle involvement is increasingly recognized as an important manifestation within the spectrum of systemic sclerosis, particularly in overlap syndromes and scleromyositis (26, 29). The pooled reduction in creatine kinase levels, combined with the clinical improvements in muscle strength reported by several cohorts, suggests meaningful therapeutic activity of IVIG in this setting (24, 26, 29). CK should, however, be interpreted as a biochemical marker of muscle injury rather than a direct measure of muscle function. Importantly, clinical improvement was also documented using muscle-strength assessments, including MRC-based measures in several cohorts.

Furthermore, studies such as that of Chaigne et al. demonstrated significant corticosteroid-sparing effects in patients treated for SSc-associated myopathy, an observation of particular importance given the well-established association between glucocorticoid exposure and scleroderma renal crisis (26). This finding should not be interpreted as supporting routine high-dose glucocorticoid therapy for systemic sclerosis itself; rather, IVIG may permit reduction of glucocorticoid exposure when glucocorticoids are required for associated inflammatory muscle disease. Therefore, therapies capable of controlling muscle inflammation while reducing corticosteroid requirements may provide substantial clinical benefit (26, 29).

An interesting aspect of the present analysis is that favorable outcomes were particularly reported in manifestations with a potentially immune-mediated component, especially SSc-associated myopathy, whereas interpretation of gastrointestinal dysmotility and skin fibrosis is more complex. The pooled effect sizes were moderate (SMDs ranging from −0.53 to −0.73). While inflammation contributes to disease initiation and progression, established fibrosis may become partially autonomous and less responsive to immunomodulation (4–6). These observations generate the hypothesis that patients with active immune-mediated manifestations may be more responsive to IVIG, but the available studies did not prospectively classify patients into validated inflammatory versus fibrotic phenotypes.

The results of this review should also be interpreted in the context of current therapeutic developments in systemic sclerosis. Over the last decade, treatment options have expanded considerably with the introduction of mycophenolate mofetil, rituximab, tocilizumab, nintedanib, and hematopoietic stem cell transplantation in selected patients (6–10). Nevertheless, a substantial proportion of patients continue to experience refractory manifestations despite these therapies. Based on the populations represented in the available studies, IVIG may occupy a niche role as an adjunctive or rescue treatment, particularly for SSc-associated inflammatory myopathy or myositis overlap, severe gastrointestinal manifestations refractory to conventional supportive and immunomodulatory approaches, inflammatory musculoskeletal involvement, or intolerance/contraindication to other therapies (23, 25, 31, 35, 38). The current evidence does not define a formal treatment sequence or establish IVIG as a replacement for approved or guideline-supported organ-specific therapies.

Available studies generally reported acceptable tolerability of IVIG, although adverse-event reporting was heterogeneous and serious events were documented, including acute kidney injury, aseptic meningitis, thrombotic events, and hematologic complications. This characteristic may be particularly relevant in systemic sclerosis, where glucocorticoid exposure is a recognized risk factor for scleroderma renal crisis (24). The corticosteroid-sparing effects observed in several cohorts suggest that IVIG may help reduce glucocorticoid exposure in selected patients treated for associated inflammatory manifestations, particularly myopathy (25, 26, 29). Although cost and accessibility remain important limitations, the available evidence supports individualized assessment of benefit and risk rather than a definitive favorable safety comparison with other systemic therapies.

The present study also provides an important contribution to the existing literature. Previous systematic reviews by Garrote-Corral et al. (32) and Koczanowski et al. (33) summarized the available evidence regarding IVIG use in systemic sclerosis but were primarily qualitative in nature. In contrast, our study provides a quantitative synthesis of the available data and estimates pooled effects across multiple clinical domains. This distinction allows a more structured assessment of the magnitude and uncertainty of the available treatment effects and provides a framework for future research (32, 33). Several limitations should be acknowledged. First, most included studies were observational, retrospective, and uncontrolled, resulting in an overall low-to-moderate certainty of evidence (19). Second, substantial heterogeneity was observed for cutaneous outcomes, likely reflecting differences in disease duration, baseline severity, treatment regimens, and concomitant therapies. Because concomitant immunosuppressive treatment was frequent, particularly with mycophenolate mofetil, glucocorticoids, cyclophosphamide, methotrexate, and other agents, the independent contribution of IVIG cannot be reliably separated from background treatment effects in many observational cohorts. Third, sample sizes were generally small, a common limitation in rare diseases such as systemic sclerosis (1, 2). Fourth, outcome definitions and follow-up durations varied considerably across studies. In addition, historical differences in SSc classification criteria across the study period may have contributed to heterogeneity in patient selection and disease-subset definitions. Finally, publication bias cannot be excluded, particularly given the tendency for positive experiences with IVIG to be reported more frequently than negative studies. The certainty of evidence according to the GRADE framework is summarized in **Table 5** (19). Formal funnel-plot asymmetry testing was not performed because fewer than 10 studies contributed to each individual pooled outcome. The review protocol was not prospectively registered, which represents an additional methodological limitation (29).

**Table 5:** GRADE summary of evidence for IVIG in systemic sclerosis.

Outcome	Studies contributing evidence	Study design	Main limitations	Certainty of evidence	Interpretation
Skin involvement assessed by mRSS	Takehara, Nacci, Poelman, Raja, Sanges, Tandaipan, Bonomi	1 RCT + observational studies	Risk of bias, heterogeneity, uncontrolled studies, concomitant therapies	Low	IVIG may reduce mRSS, particularly with repeated cycles, but confidence is limited.
Gastrointestinal involvement	Raja, Tandaipan, Mendoza, Matsuda	Observational studies/case series	Small samples, no randomized comparator, heterogeneous GI outcomes	Very low to low	IVIG may improve selected GI manifestations, especially severe dysmotility/dysphagia, but evidence remains weak.
Myopathy/muscle involvement	Chaigne, Raja, Sanges, Tandaipan	Observational studies	Confounding by corticosteroids and immunosuppressants, heterogeneous definitions of myopathy	Low	IVIG may improve muscle symptoms and CK and may have corticosteroid-sparing benefit.
Pulmonary function	Poelman, Sanges, Chaigne, Tandaipan	Observational studies	Mostly secondary outcomes, no consistent improvement, confounding	Very low	IVIG appears associated mainly with stabilization rather than clear pulmonary improvement.
Corticosteroid-sparing effect	Chaigne, Tandaipan	Observational studies	Non-randomized allocation and confounding by indication	Very low to low	Possible corticosteroid-sparing effect, particularly in SSc-associated myopathy.
Safety/adverse events	Takehara, Poelman, Sanges, Chaigne, Tandaipan and smaller series	Mixed designs	Retrospective reporting, small samples, variable follow-up	Low	IVIG was generally well tolerated; serious events were uncommon but included hemolysis, renal injury, thrombotic events and aseptic meningitis.

CK, creatine kinase, GIT, gastrointestinal tract, GRADE, Grading of Recommendations Assessment, Development and Evaluation, IVIG, intravenous immunoglobulin, mRSS, modified Rodnan Skin Score, SSc, systemic sclerosis, UCLA SCTC GIT 2.0, University of California Los Angeles Scleroderma Clinical Trial Consortium Gastrointestinal Tract Instrument version 2.0.

Studies listed in the GRADE assessment contributed evidence to either the quantitative synthesis, the qualitative synthesis, or both. Consequently, the studies included in individual meta-analyses may differ from those considered in the overall certainty-of-evidence assessment.

Despite these limitations, the present study possesses important strengths. A comprehensive search strategy was employed across multiple databases, and data were synthesized using contemporary meta-analytic methods (16, 17). The inclusion of studies evaluating diverse clinical manifestations allowed a broader assessment of IVIG efficacy across the systemic sclerosis spectrum. Furthermore, the inclusion of independent cohorts from different settings allowed assessment of the reproducibility and limitations of the observed associations across heterogeneous populations (20–30).

Future research should focus on adequately powered randomized controlled trials evaluating specific clinical phenotypes of systemic sclerosis. Particular attention should be directed toward patients with inflammatory myopathy, severe gastrointestinal involvement, and early diffuse cutaneous disease, as these clinical subsets were frequently represented among studies reporting favorable outcomes and merit prospective evaluation (24, 26–29). Standardization of outcome measures and reporting methods will also be essential to improve the quality of future evidence (16, 17).

## Conclusion

This systematic review and meta-analysis demonstrated that IVIG is associated with potential clinical benefits across multiple manifestations of systemic sclerosis. Quantitative synthesis revealed associations with improvements in skin fibrosis, gastrointestinal involvement, and inflammatory myopathy, supporting a potential adjunctive role for IVIG in selected patients. However, these findings should be interpreted cautiously because the evidence is predominantly derived from observational studies, and the only randomized placebo-controlled trial did not demonstrate significant short-term improvement in skin involvement at its primary endpoint.

Among the evaluated manifestations, favorable outcomes were observed in gastrointestinal disease and inflammatory myopathy, whereas the pooled analysis suggested a moderate effect on skin fibrosis but showed substantial heterogeneity. These findings suggest that IVIG may warrant further investigation in patients with active immune-mediated manifestations, particularly SSc-associated inflammatory myopathy, although the available evidence does not establish which clinical phenotypes are most likely to benefit. The broad spectrum of activity observed across different organ systems is consistent with the concept that IVIG exerts pleiotropic immunomodulatory effects capable of influencing several pathogenic pathways involved in systemic sclerosis.

Although the current evidence base is limited by the predominance of observational studies, small sample sizes, and heterogeneous treatment protocols, the available studies provide a rationale for further controlled investigation of IVIG in selected manifestations of SSc. Importantly, IVIG demonstrated generally acceptable tolerability, although serious adverse events were reported, and may offer a glucocorticoid-sparing approach in selected patients with systemic sclerosis-associated inflammatory myopathy, where reducing glucocorticoid exposure is clinically desirable.

Taken together, these findings do not establish IVIG as a standard treatment for systemic sclerosis but support further evaluation of its potential adjunctive role, particularly in SSc-associated inflammatory myopathy or myositis overlap and severe gastrointestinal manifestations refractory to conventional management. Future adequately powered randomized controlled trials are required to define its efficacy, optimal timing, dosing regimens, duration of therapy, and patient populations most likely to benefit from treatment. Until such data become available, the present study provides a quantitative synthesis of the currently available evidence and highlights the need for standardized controlled trials to clarify the clinical role of IVIG in systemic sclerosis.

## Data Availability

The original contributions presented in the study are included in the article/supplementary material. Further inquiries can be directed to the corresponding authors.
